# Relationship between fraction of exhaled nitric oxide and airway morphology assessed by three-dimensional CT analysis in asthma

**DOI:** 10.1038/s41598-017-10504-w

**Published:** 2017-08-31

**Authors:** Koji Nishimoto, Masato Karayama, Naoki Inui, Kazutaka Mori, Masato Kono, Hironao Hozumi, Yuzo Suzuki, Kazuki Furuhashi, Noriyuki Enomoto, Tomoyuki Fujisawa, Yutaro Nakamura, Hiroshi Watanabe, Takafumi Suda

**Affiliations:** 1 Second Division, Department of Internal Medicine, Hamamatsu University School of Medicine, 1-20-1 Handayama, Hamamatsu, 431-3192 Japan; 2 Department of Clinical Pharmacology and Therapeutics, Hamamatsu University School of Medicine, 1-20-1 Handayama, Hamamatsu, 431-3192 Japan

## Abstract

Fraction of exhaled nitric oxide (FeNO) provides information about chronic inflammation in asthma. However, its relationship with structural changes in the airways is unknown. We aimed to evaluate the correlation between computer-based airway changes and FeNO in patients with asthma. The wall area (WA) and airway inner luminal area (Ai) of the third- to sixth-generation bronchi were measured using three-dimensional computed tomography in asthmatic patients. Each value was corrected by body surface area (BSA). Relationships between FeNO and WA/BSA and Ai/BSA were evaluated. Forty-one clinically stable patients with asthma were evaluated. FeNO was significantly correlated with WA/BSA of the third-, fourth-, fifth- and sixth-generation bronchi (Spearman correlation coefficient (*ρ*) = 0.326, *p* = 0.041; *ρ* = 0.356, *p* = 0.025; *ρ* = 0.496, *p* = 0.002; and *ρ* = 0.529, *p* < 0.001, respectively). The correlation with sixth-generation bronchi was significantly greater than with the third-generation bronchi (*p* = 0.047). Partial rank correlation analysis indicated FeNO was significantly correlated with WA/BSA of the sixth-generation bronchi, independent from confounding factors of Ai/BSA, age, duration of asthma, dose of inhaled corticosteroid, blood eosinophil percentage, and blood IgE (*ρ* = 0.360, *p* = 0.034). In contrast, there was no correlation between FeNO and Ai/BSA. FeNO correlates with bronchial wall thickening in asthma patients. Measurement of FeNO may be useful to detect airway remodeling in asthma.

## Introduction

Asthma is an airway inflammatory disease characterized by airway narrowing and wall thickening^1^. Spirometry and peak expiratory flow measurements are widely used for the diagnosis and management of asthma^1^. These physiological function tests evaluate the severity of airflow limitation caused by airway narrowing, but cannot directly evaluate bronchial wall thickening, which is an important pathogenic feature in asthma^2, 3^.

Bronchial wall thickening in airway remodeling is attributed to the infiltration of inflammatory cells, thickening of the basement membrane, epithelial hyperplasia, and smooth muscle hypertrophy^4–7^. These findings are mainly based on postmortem studies because of the difficulty in sampling bronchial tissues in clinical practice. Some previous studies using transbronchial biopsy reported similar pathologic changes in the airways of asthmatic patients, which correlated with bronchial hyperresponsiveness and disease severity^8–11^. In addition to being an invasive procedure, endoscopic biopsy is limited because it can only sample from the surface of bronchial tissue and therefore cannot evaluate all layers of the bronchial wall and the wide range of bronchial trees.

With recent advances in imaging technology, quantitative computed tomography (CT) analysis can provide detailed information about airway changes in lung diseases^12–19^. CT analysis can assess the airway inner luminal area and total wall area in a wide range of airway trees in a non-invasive manner that cannot be assessed by endoscopic biopsy. We previously evaluated the airways of third- to sixth-generation bronchi in patients with chronic obstructive pulmonary disease using three-dimensional CT analysis and reported correlations between morphological changes of the airway and physiological function tests^20^. Regarding asthmatic patients, airway changes by chest CT were correlated with pulmonary function^3, 21^, hyperresponsiveness^22^, and disease severity^23–28^. Bronchial wall thickening assessed by CT also demonstrated a correlation with pathologic measurements of remodeling in transbronchial biopsy specimens from asthmatic patients^27, 29, 30^.

Chronic airway inflammation is an essential component of asthma, which leads to airway remodeling. The fraction of exhaled nitric oxide (FeNO) is considered a marker of airway inflammation^31–33^ and its measurement provides supplemental information for the diagnosis and management of asthma in a simple and noninvasive manner. FeNO correlates with symptoms^34, 35^, physiological function tests^36, 37^, and disease severity^35, 36^ and FeNO measurement-based therapy for asthma demonstrated favorable clinical outcomes^38, 39^. However, how and whether structural changes in the airways in asthma correlate with FeNO is unknown.

The current study aimed to evaluate the relationship between FeNO and airway structure assessed by three-dimensional CT in patients with asthma.

## Results

### Patient characteristics

The characteristics of the patients are shown in Table 1. The study population was predominantly middle-aged and older adults with median age of 68 years. Twenty-five patients (61.0%) were female. The median duration of asthma was 19 years and 29 patients had disease duration of more than 10 years. The median (range) blood eosinophil percentage was 5.3 (0.5–21.1) %, and the median blood total IgE was 216 (9–1600) IU/mL. Most patients received inhaled corticosteroids. Six (14.6%), 23 (56.1%), and 10 (24.4%) patients received low- (<250 μg), medium- (250–500 μg) and high-dose (>500 μg) of inhaled corticosteroids at fluticasone equivalent, respectively.

**Table 1:** Patient characteristics.

	All patients (n = 41)
Age, years	68 (16–80)
Sex; female	25 (61.0)
Duration of asthma, years	19 (1–72)
BMI, kg/m^2^	22.7 (16.0–35.3)
Blood %eosinophil, %	5.3 (0.5–21.0)
Blood total IgE, IU/mL	216 (9–1600)
FeNO, ppb	27.0 (8.2–329)
FEV_1_, % predicted	87.9 (46.3–120.0)
FEV_1_/FVC ratio, %	71.6 (55.1–92.7)
MMF, % predicted	43.4 (11.9–135)
GINA treatment steps 1/2/3/4/5	2 (4.9)/4 (9.8)/6 (14.6)/27 (65.8)/2 (4.9)
Inhaled corticosteroid	39 (95.1)
Inhaled corticosteroid dose, μg^§^	400 (100–1200)
Long-acting beta 2 agonist	26 (63.4)
Leukotriene receptor antagonist	21 (51.2)

Data are expressed as number (%) or median (range). BMI, body mass index; FeNO, fraction of exhaled nitric oxide; FVC, forced vital capacity; FEV_1_, forced expiratory volume in 1 second; MMF, maximum mid-expiratory flow rate; GINA, Global Initiative for Asthma. ^§^Among 39 patients receiving inhaled corticosteroids.

### Fraction of exhaled nitric oxide

The median concentration of FeNO was 27.0 (8.2–329.4) ppb. FeNO was significantly correlated with age (Spearman correlation coefficient (ρ) = 0.370, *p* = 0.017), but not with duration of asthma, body mass index, blood eosinophil percentage, blood total IgE, percent predicted FEV_1_, or dose of inhaled corticosteroid (Table 2).

**Table 2:** Correlations between fraction of exhaled nitric oxide and clinical parameters.

	ρ	*p*-value
Age	0.370	0.017
Duration of asthma, years	−0.137	0.391
BMI	0.014	0.929
Blood %eosinophil	0.140	0.396
Blood total IgE	0.022	0.895
FEV_1_, % predicted	−0.033	0.728
FEV_1_/FVC ratio, %	−0.237	0.136
MMF, % predicted	−0.156	0.330
Inhaled corticosteroid dose, μg	0.090	0.586

Data are expressed as the Spearman rank correlation coefficient (ρ) and *p*-value. BMI, body mass index; FVC, forced vital capacity; FEV_1_, forced expiratory volume in 1 second; MMF, maximum mid-expiratory flow rate.

### Three-dimensional CT analysis of the airways

The wall area (WA) corrected by body surface area (BSA, WA/BSA) and airway inner luminal area (Ai) corrected by BSA (Ai/BSA) from the third- to sixth-generation bronchi assessed by three-dimensional CT are shown in Table 3. The WA/BSA was significantly correlated with Ai/BSA in the fourth-, fifth- and sixth-generation bronchi (ρ = 0.372, *p* = 0.018; ρ = 0.376, *p* = 0.018; and ρ = 0.384, *p* = 0.016, respectively), but not in the third-generation (ρ = 0.217, *p* = 0.180). The WA/BSA was significantly correlated with age in the third-, fourth-, fifth- and sixth-generation bronchi (ρ = 0.566, *p* < 0.001; ρ = 0.533, *p* < 0.001; ρ = 0.562, *p* < 0.001; and ρ = 0.556, *p* < 0.001, respectively, Table 4), but not with duration of asthma, blood eosinophil percentage, blood total IgE, percent predicted FEV_1_, or dose of inhaled corticosteroid. The Ai/BSA was moderately correlated with blood eosinophil percentage in the third- and fifth-generation bronchi (ρ = 0.385, *p* = 0.017; and ρ = 0.380, *p* = 0.020, respectively, Table 5), but not with the other clinical factors.

**Table 3:** Three-dimensional CT analysis of the airway morphology.

	Third-generation bronchi	Fourth-generation bronchi	Fifth-generation bronchi	Sixth-generation bronchi
Ai, mm^2^	28.7 (14.2–49.7)	7.31 (4.27–18.4)	4.82 (1.88–10.9)	2.90 (1.13–6.53)
Ai/BSA, mm^2^/m^2^	19.6 (8.75–31.0)	4.89 (2.57–11.0)	3.02 (1.09–6.52)	1.76 (0.66–4.08)
WA, mm^2^	36.3 (28.2–44.3)	29.2 (16.4–36.3)	25.0 (21.0–33.6)	22.0 (17.9–31.7)
WA/BSA, mm^2^/m^2^	23.1 (17.3–30.1)	18.8 (10.6–25.4)	16.0 (12.5–20.5)	14.1 (11.4–19.8)

Data are expressed as the median (range). Ai, airway inner luminal area; WA, wall area; BSA, body surface area.

**Table 4:** Correlations between airway wall areas adjusted for body surface area and clinical parameters.

	Third-generation bronchi		Fourth-generation bronchi		Fifth-generation bronchi		Sixth-generation bronchi	
	ρ	*p*-value	ρ	*p*-value	ρ	*p*-value	ρ	*p*-value
Age	0.566	<0.001	0.533	<0.001	0.562	<0.001	0.556	<0.001
Duration of asthma	0.057	0.725	0.182	0.260	−0.112	0.496	−0.149	0.367
Blood %eosinophil	0.005	0.977	0.169	0.311	0.071	0.675	0.146	0.390
Blood total IgE	−0.197	0.250	−0.204	0.233	−0.066	0.705	−0.177	0.310
FEV_1_, % predicted	0.056	0.734	0.099	0.544	0.202	0.217	0.174	0.289
FEV_1_/FVC ratio	−0.156	0.336	−0.219	0.174	−0.198	0.226	−0.192	0.240
MMF, % predicted	−0.089	0.585	−0.086	0.597	−0.073	0.659	−0.060	0.715
Inhaled corticosteroid dose	−0.141	0.400	−0.014	0.933	−0.184	0.268	−0.269	0.102

Data are expressed as Spearman rank correlation coefficient (ρ) and *p*-value. FVC, forced vital capacity; FEV_1_, forced expiratory volume in 1 second; MMF, maximum mid-expiratory flow rate.

**Table 5:** Correlations between airway inner luminal areas adjusted for body surface area and clinical parameters.

	Third-generation bronchi		Fourth-generation bronchi		Fifth-generation bronchi		Sixth-generation bronchi	
	ρ	*p*-value	ρ	*p*-value	ρ	*p*-value	ρ	*p*-value
Age	0.060	0.715	−0.035	0.829	−0.016	0.921	0.019	0.910
Duration of asthma	0.165	0.310	0.142	0.382	0.025	0.881	−0.042	0.798
Blood %eosinophil	0.385	0.017	0.225	0.174	0.380	0.020	0.278	0.095
Blood total IgE	−0.034	0.843	0.031	0.856	0.137	0.433	0.149	0.394
FEV_1_, % predicted	0.142	0.383	0.204	0.207	0.137	0.406	0.202	0.217
FEV_1_/FVC ratio	0.087	0.595	0.162	0.318	0.207	0.206	0.247	0.129
MMF, % predicted	0.152	0.348	0.211	0.192	0.184	0.263	0.242	0.137
Inhaled corticosteroid dose	0.150	0.368	0.080	0.631	0.073	0.664	−0.080	0.635

Data are expressed as Spearman rank correlation coefficient (ρ) and *p*-value. FVC, forced vital capacity; FEV_1_, forced expiratory volume in 1 second; MMF, maximum mid-expiratory flow rate.

### Correlations between fraction of exhaled nitric oxide and three-dimensional CT analysis of the airways

FeNO was significantly correlated with WA/BSA of the third-, fourth-, fifth- and sixth-generation bronchi (ρ = 0.326, *p* = 0.041; ρ = 0.356, *p* = 0.025; ρ = 0.496, *p* = 0.002; and ρ = 0.529, *p* < 0.001, respectively, Table 6). The magnitude of the correlation demonstrated a gradual increase from the third- toward the sixth-generation bronchi, and was statistically more significant in sixth-generation bronchi compared with third-generation bronchi (*p* = 0.047, Table 7). In contrast, FeNO was not correlated with Ai/BSA of the third- to sixth-generation bronchi (Table 6).

**Table 6:** Correlations between fraction of exhaled nitric oxide and airway structures

	Spearman rank correlation		Partial rank correlation*	
	ρ	*p*-value	ρ	*p*-value
Ai/BSA				
Third-generation bronchi	0.108	0.507	0.032	0.854
Fourth-generation bronchi	0.067	0.680	0.027	0.878
Fifth-generation bronchi	0.144	0.380	0.050	0.779
Sixth-generation bronchi	0.205	0.209	0.113	0.520
WA/BSA				
Third-generation bronchi	0.326	0.041	0.116	0.508
Fourth-generation bronchi	0.356	0.025	0.154	0.378
Fifth-generation bronchi	0.496	0.001	0.272	0.114
Sixth-generation bronchi	0.529	<0.001	0.360	0.034

Data are expressed as the correlation coefficient (ρ) and *p*-value. *Partial rank correlations between fraction of exhaled NO and Ai/BSA were adjusted for WA/BSA, age, duration of asthma, dose of inhaled corticosteroid, percentage blood eosinophil, and total blood IgE, and those between fraction of exhaled NO and WA/BSA were adjusted for Ai/BSA and the other five parameters. Ai, airway inner luminal area; WA, wall area; BSA, body surface area.

**Table 7:** Differences in the magnitude of correlations between airway wall areas and fraction of exhaled nitric oxide among bronchial generations.

	Δρ	*p*-value
Third–fourth	−0.030	0.767
Third–fifth	−0.140	0.131
Third–sixth	−0.203	0.047
Fourth–fifth	−0.140	0.108
Fourth–sixth	−0.173	0.054
Fifth–sixth	−0.033	0.612

Data are expressed as the difference in Spearman rank correlation coefficient (Δρ) and p-value by the Meng-Rosenthal-Rubin method. 3rd, third-generation bronchi; 4th, fourth-generation bronchi; 5th, fifth-generation bronchi; 6th, sixth-generation bronchi.

Partial rank correlation analysis was performed to assess the correlation between FeNO and three-dimensional CT parameters by eliminating potential confounding clinical factors. FeNO was significantly correlated with WA/BSA of the sixth-generation bronchi (but not with those of the third- to fifth-generation bronchi) when adjusted for age, duration of asthma, blood eosinophil percentage, blood total IgE, dose of inhaled corticosteroid, and Ai/BSA (ρ = 0.360, *p* = 0.034, Table 6). Conversely, FeNO was not significantly correlated with Ai/BSA of the third- to sixth-generation bronchi when adjusted for the same clinical factors and WA/BSA (Table 6).

## Discussion

In the current study, FeNO was correlated with the thickness of wall area throughout the third- to sixth-generation bronchi in asthma patients, and the correlation was strongest in the area of the sixth-generation bronchi. Partial rank correlation analysis demonstrated a significant correlation between FeNO level and the wall area of sixth-generation bronchi, independent of clinical factors that might affect FeNO expression and/or morphological airway changes. Therefore, the measurement of FeNO might be useful to assess bronchial wall thickening in asthma.

Bronchial wall thickening is attributed to increased epithelium, smooth muscle, and submucosa, and is accompanied by the infiltration of inflammatory cells^4–7^. Eosinophils are a major airway inflammatory cell subset that produces exhaled NO by the expression of inducible nitric oxide synthase^7, 31, 33^. It was reported that FeNO concentration was correlated with eosinophilic airway inflammation in mucosal biopsies^32^. Mahut *et al*. reported that FeNO was correlated with subepithelial eosinophilic infiltration and reticular basement membrane thickening in bronchial mucosal biopsies of 28 asthmatic children^37^. Eosinophilic inflammation plays an important role in the airway remodeling of asthma^40–42^, and might explain the correlation between FeNO and bronchial wall thickening as assessed by three-dimensional CT in the current study. In addition to the increased production of NO, the diffusion capacity of airway NO into the surrounding blood vessels also affects the levels of FeNO^43, 44^. Wall thickening of the airways may inhibit the diffusion of airway NO, which may also contribute to the correlation between bronchial wall area and FeNO. Several clinical factors might affect the bronchial wall area changes and/or FeNO expression including age, duration of asthma, and the use of inhaled corticosteroids^21, 28, 45, 46^. Airway narrowing, another key pathogenetic characteristic of asthma, was also correlated with wall thickening of the fourth-, fifth- and sixth-generation bronchi. Using partial rank correlation analysis, we demonstrated significant correlations between FeNO and wall thickening even after the exclusion of potential confounding factors.

The serial assessment of the bronchial tree revealed differences in the correlation between FeNO and wall thickening among bronchial generations. De Blic *et al*. reported a correlation between NO production and semiquantitative scores of bronchial wall thickness obtained from the high resolution CT scans of 37 children with severe asthma (ρ = 0.45, *p* = 0.02). However, they did not evaluate the actual thickness of each bronchial wall, but only counted the total number of visible bronchi with no distinction of bronchial generations in five sections of horizontal CT images^29^. In the current study using three-dimensional CT analysis of the bronchial trees, the correlation between FeNO and wall area gradually increased toward the sixth-generation bronchi, and was strongest in the sixth-generation bronchi. Although the underlying reason for the difference in the correlation with FeNO among bronchial generations is unknown, different parts of the bronchial tree might unevenly contribute to FeNO. Aikawa *et al*. reported a postmortem analysis where patients who died of asthma attack had significantly severe goblet hyperplasia in restricted airways compared with asthmatic patients that died of non-asthma related causes. The change was only observed in the peripheral airways (defined by the absence of cartilage), but not in the central airways^6^. Hamid *et al*. reported that airways with an inner luminal diameter <2 mm had significantly larger numbers of activated eosinophils than those with a >2 mm in lung specimens obtained from asthmatic patients who underwent surgical resection for lung cancer^7^. These data suggested that peripheral airways undergo greater inflammatory changes than the central airways in asthma. Although we could not evaluate more than sixth-generation bronchi in the current study, the gradual increase in the magnitude of correlation between FeNO and wall area toward the sixth-generation bronchi suggests that the more distal airways probably have a stronger influence on FeNO.

Age was correlated with both bronchial wall area and FeNO. Bai *et al*. evaluated the pathologic changes in the airways of 27 fatal asthmatic patients. The subjects were in their 40 s (n = 13) and had significantly greater bronchial wall areas, compared with younger subjects (n = 14, range 17–23 years), which was mainly because of an increase in smooth muscle area^9^. Gelb *et al*. evaluated FeNO in 106 non-smoking healthy subjects and reported that elderly subjects ≥60 years had significantly higher FeNO levels (median of 27 ppb) than younger subjects <60 years (median of 21 ppb, p = 0.006). They suggested that the potential mechanism for increased FeNO with age was caused by a reduction in the diffusing capacity of NO into surrounding vessels^47^. The correlation between FeNO and bronchial wall area was independent from age in the current study, although age-related changes should be considered when interpreting the value of FeNO in clinical practice.

The current study had three main limitations. First, we evaluated the six segmental bronchi (B1, B2, B3, B8, B9, and B10) in the right lung. Middle-lobe bronchi as well as B6 and B7 were excluded from analysis because they are readily associated with atelectasis, airway collapse or mucus hypersecretion. It is assumed that the artifact caused by airway collapse can be avoided by using this method^19, 20^. Actually, two patients had atelectasis and airway collapse in the middle lobe. The left lung was also excluded from analysis to avoid artifacts caused by transmitted cardiac motion. Few study directly compare the morphological changes of right lung and those of left lung in relationship with eosinophilic inflammation or FeNO. The optimal method to assess airway morphology should be further investigated. Second, the limited resolution of CT did not allow a detailed evaluation of distal airways greater than sixth-generation bronchi. There are two compartments of FeNO that represent different parts of the airways. Central airway NO represents airways of the first- to sixteenth-generation bronchi, whereas peripheral airway/alveolar NO represents airways of the seventeenth- to twenty-third-generation bronchi^48^. The evaluation of small airways is needed to obtain further information regarding alveolar NO. Third, the elevation of FeNO was not fully accountable by bronchial wall thickening alone. Wall thickening is a result of several pathologic changes including epithelial hyperplasia and increased smooth muscle, of which eosinophilic inflammation is but one of the causes^4, 6–9^. In the current study, FeNO was not correlated with Ai/BSA, which might be because these factors including eosinophilic inflammation had strong effect on wall area than airway intraluminal area. In addition, several clinical factors affect FeNO including inhaled corticosteroids (administered to most of the study patients), or smoking status (only never- or light former-smokers were included in the study), which affects the level of exhaled NO^46^. Even though there was no significant correlation between FeNO and these factors in the current study, the potential influence should be considered when interpreting FeNO. In particular, inhaled corticosteroids decrease both FeNO level and bronchial wall thickening in asthma^39, 44, 45, 49^, which could affect the results. However, our data demonstrated that correlation between FeNO and bronchial wall thickening was independent from the dose of inhaled corticosteroid in partial correlation analysis. Thus, our data might be applicable in clinical practice among asthma patients receiving inhaled corticosteroids.

In conclusion, FeNO was correlated with bronchial wall thickening from the third- to sixth-generation bronchi in asthma patients. The measurement of FeNO may be useful when assessing structural changes in the airways of asthma patients.

## Methods

### Subjects

Clinically stable asthmatic patients were enrolled in this study. The patients were required to satisfy the definition of asthma set by the Global Initiative for Asthma (GINA)^1^. Exclusion criteria were: uncontrolled asthma defined by GINA^1^; requirement of treatment change, respiratory tract infection, or exacerbation (defined as episodes of a progressive increase in dyspnea, coughing, wheezing, or chest tightness, or a combination of these symptoms^1^) within 4 weeks before enrollment; current or former smokers with smoking history ≥10 pack-years; use of oral corticosteroid in the previous 6 months; use of long-term oxygen therapy; diffuse lung diseases; neuromuscular diseases; presence of congenital anomalies of the bronchial tree; and a history of thoracic surgery. The study was conducted in accordance with the ethical standards of the Declaration of Helsinki and approved by the Institutional Review Board of Hamamatsu University School of Medicine (Hamamatsu, Japan). Each patient provided written informed consent to be included in the study.

### Three-dimensional CT analysis of the airways

**Figure 1:**
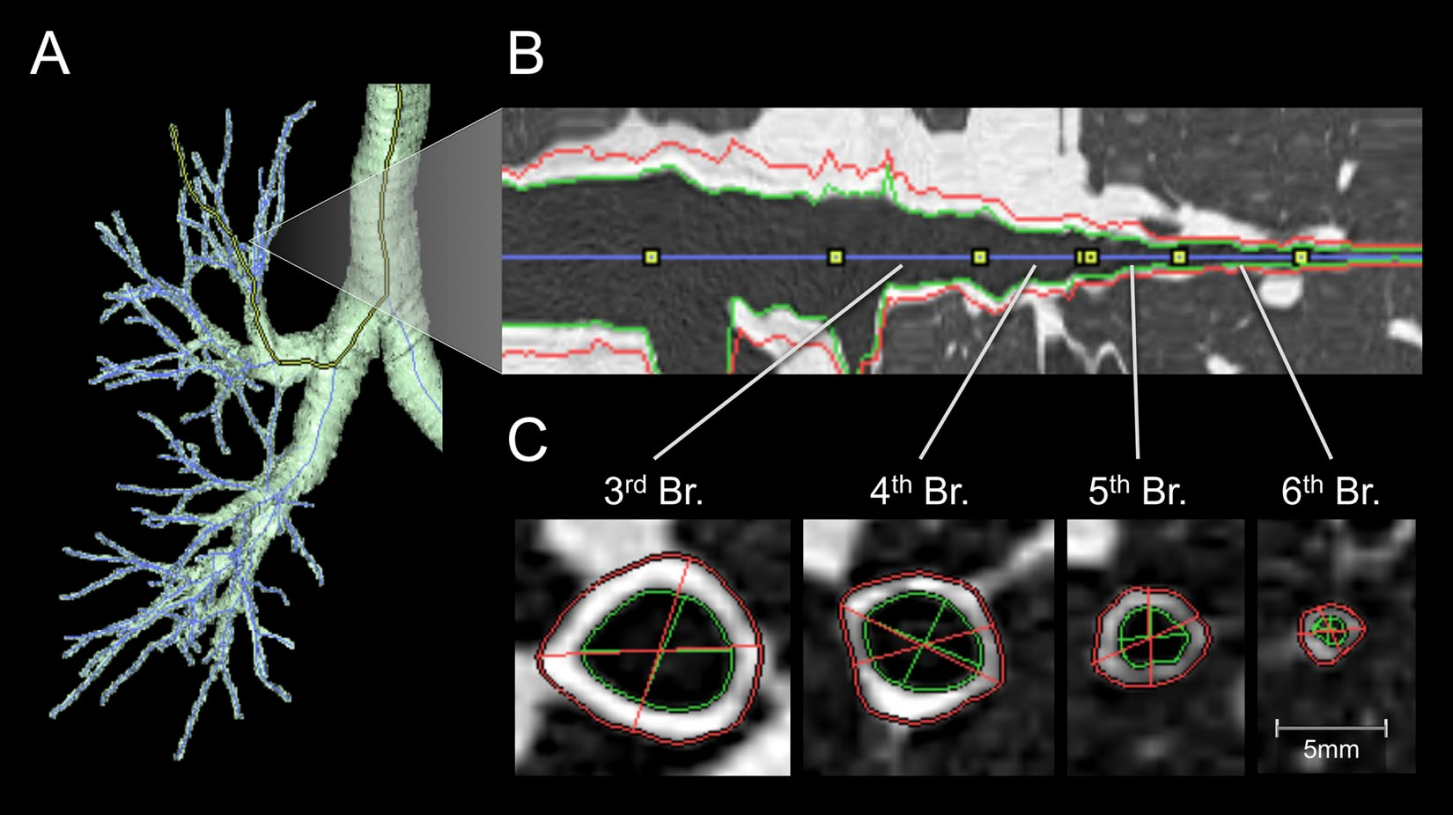
Three-dimensional CT analysis of the airways. (**A**) Three-dimensional image of the airway trees. (**B**) Longitudinal image of the reformatted airway. (**C**) Short axis image of the airway. Red and green lines indicate the outer and inner edges of the airways, respectively. The areas within the green line and between the red and green lines are the airway inner luminal area and wall area, respectively.

Multidetector-row CT (MDCT) imaging was performed using a 64-slice MDCT machine (Aquilion-64; Toshiba Medical Systems, Tokyo, Japan) in the supine position at full inspiration breath-hold for research purpose. The details of MDCT scanning were described a previous study^20^. We obtained three-dimensional CT images of airway trees reconstructed from the MDCT images using image-analyzing software (SYNAPSE VINCENT; Fuji Film, Tokyo, Japan). All airway candidates were detected using the airway classifiers learned by machine learning. Based on the detected airway candidates, an airway center line was constructed using a minimum spanning tree, and inner and outer airway contours were automatically extracted using the graph cuts method^50^. A bronchial pathway was reconstructed into multiplanar reconstruction images with a window width of 1600 HU and a window level of −600 HU (Fig. 1). If an error occurred during the automatic extraction of airway contours, then it was corrected manually. Measurements of airways were performed in the six bronchi (B1, B2, B3, B8, B9, and B10) in the right lung according to methods reported previously^19, 20^ and the analysis was performed by one of the authors (KM) who was blinded to patient’s information. For each selected bronchus, four levels of the airways (third- (segmental), fourth- (sub-segmental), fifth-, and sixth-generation bronchi) were identified by tracing airway trees peripherally on the same trunk of each segmental bronchus. At the midpoint of each level of airway, WA and Ai perpendicular to the long axis of the airway were computed automatically (Fig. 1). The WA was defined as the area between the outer and inner edge of the airway. Six images in each level of the bronchus and a total of 24 images in each subject were evaluated, and the results of bronchi from each generation were expressed separately as the mean of six airways. To eliminate the potential influence of body size of each patient, the Ai and WA were adjusted by BSA (Ai/BSA and WA/BSA, respectively), according to previous reports^3, 12, 15, 21, 25^.

### Measurement of fraction of exhaled nitric oxide

FeNO was measured with an online method at a flow of 50 mL/s using a chemiluminescence NO analyzer, Sievers NOA280i (GE Analytical Instruments, Boulder, Colorado, USA) according to American Thoracic Society/European Respiratory Society recommendations^51^. The measurements were performed in triplicate, and the results were reported as the mean of three measurements. All subjects abstained from food and coffee for 2 h, and alcohol for 12 h before testing.

### Measurement of spirometry

An Autospirometer System 7 (Minato Medical Science Co., Ltd., Osaka, Japan) was used to measure spirometry according to the standards of the Japanese Respiratory society^52^. Spirometry and measurements of FeNO were performed on the same day as the chest CT scans, and spirometry was performed after measurement of FeNO.

### Statistical analysis

Correlation between continuous variables was undertaken using the Spearman rank correlation coefficient. Partial rank correlation analysis between FeNO and WA/BSA and Ai/BSA were performed with adjustments for age, duration of asthma, dose of inhaled corticosteroid, percentage of blood eosinophils, and blood total IgE as the potential confounding factors^53, 54^. Differences between rank correlation coefficients were evaluated by the Meng-Rosenthal-Rubin method^55^. Data are the median (range) unless indicated otherwise. Statistical tests were two-sided, and *p* < 0.05 was considered significant. Values were analyzed using JMP v9.0.0 software (SAS Institute Japan, Tokyo, Japan), except for partial rank correlation analysis and Meng-Rosenthal-Rubin method which used R version 3.2.4 (The R Foundation for Statistical Computing, Vienna, Austria, 2016) with additional packages (cocor, psych)^53–55^.
